# Body Size Regression Formulae, Proximate Composition and Energy Density of Eastern Bering Sea Mesopelagic Fish and Squid

**DOI:** 10.1371/journal.pone.0132289

**Published:** 2015-08-19

**Authors:** Elizabeth H. Sinclair, William A. Walker, James R. Thomason

**Affiliations:** National Marine Mammal Laboratory, Alaska Fisheries Science Center, National Marine Fisheries Service, National Oceanic Atmospheric Administration, Seattle, Washington, United States of America; The Evergreen State College, UNITED STATES

## Abstract

The ecological significance of fish and squid of the mesopelagic zone (200 m–1000 m) is evident by their pervasiveness in the diets of a broad spectrum of upper pelagic predators including other fishes and squids, seabirds and marine mammals. As diel vertical migrators, mesopelagic micronekton are recognized as an important trophic link between the deep scattering layer and upper surface waters, yet fundamental aspects of the life history and energetic contribution to the food web for most are undescribed. Here, we present newly derived regression equations for 32 species of mesopelagic fish and squid based on the relationship between body size and the size of hard parts typically used to identify prey species in predator diet studies. We describe the proximate composition and energy density of 31 species collected in the eastern Bering Sea during May 1999 and 2000. Energy values are categorized by body size as a proxy for relative age and can be cross-referenced with the derived regression equations. Data are tabularized to facilitate direct application to predator diet studies and food web models.

## Introduction

Mesopelagic (200 m–1000 m depth) fishes and cephalopods play a central role in marine ecology as vertically migrating planktivores and principal prey to a wide range of top predators [1,2]. It is widely recognized that the biomass of mesopelagic micronekton is greatly underestimated due to limitations in catching them [2]. Even so, the biomass of mesopelagic fishes alone is estimated to exceed that of worldwide commercial fish catches [3,4]. Their great biomass, diel vertical migration from ocean depths, high consumption of zooplankton and ubiquity in upper pelagic predator diets indicates significant carbon capture and energy transferal by mesopelagic micronekton throughout the water column [5,6,7] resulting in a prominent contribution to the surface-to-depth nutritional circulation (‘biological pump’) of the world oceans [8, 9]. Therefore, assessing the size-related energetic value of dominant species of the mesopelagic zone is relevant to interpreting the ecological linkages and dynamics of the pelagic system as a whole.

Increasing research focus on the structure of the marine food web underscores the need for detailed information on prey size and energetic value. Prey length and weight are primary variables in calculations of biomass consumption by individual predators and also indicate broader ecological patterns of predator foraging location and habitat use, since spatial distribution varies with age/body size for many marine species [10,11]. Therefore, the size-related energetic value of prey can point to the energetic potential of different foraging depths or regions specific to predator foraging habitat [12]. Prey size distribution and biomass consumption estimates are also integral to deciphering the trophic interactions and structure of marine communities through the use of ecosystem models [13], and bio-energetic modeling relies on specific values to project realistic ecosystem profiles [14]. Accordingly, estimates of the size and energetic value of prey serve as baseline values in models applied to ecosystem management [15].

In the absence of whole remains, the measurement of fish sagittal otoliths and squid beaks are long-standing tools to estimate prey size [16–20]. The body size reconstruction of fish based on otolith measurement has become progressively available for mesopelagic [21–26] and benthy-mesopelagic species [27,28] of the world’s oceans. Length and weight regressions based on beak measurements have been developed for numerous families of squid [29,30], particularly the Gonatidae which frequent the mesopelagic [30,31,27].

The proximate composition and energetic value of mesopelagic fish and squid has been extensively researched in the Gulf of Mexico [12], the southwest Atlantic Ocean [32] and Antarctica [33–36] but, less so in the northeastern North Pacific Ocean or its associated waters. Research there has focused on the energetics of epipelagic and benthic species important in the diet of marine mammals and birds [37–40], but not on the mesopelagic fishes and cephalopods that transit between zones.

In this study, we develop regression formulae to determine the length and weight of mesopelagic fish and squid based on otolith and beak measurements from species that dominated our directed catch in the southeastern Bering Sea. We then evaluate the proximate contribution of fat and protein to their energetic potential relative to body size. Our findings are provided in tabular format intended for direct application to diet studies of higher trophic level predators, and to growing efforts towards refining the details of ecosystem modeling.

## Materials and Methods

### Length-weight regression analyses

We developed length-weight regression analyses for 19 species of fish and 13 species of squid that dominated catch numbers in a dedicated mesopelagic survey effort in the eastern Bering Sea, May 1999 and 2000 [41]. Fish regressions were developed between otolith length (OL) or height (OH) and standard length (SL) and weight (WT) using measurements from either left- or right-sided otoliths. Otolith length is the greatest distance between anterior and posterior otolith margins and OH is the greatest distance from the ventral to the dorsal otolith margin [23]. Dentary anterior tooth length (DATL) was used in the case of (*Chauliodus macouni*), in lieu of measuring the very tiny otoliths typical of the Stomiidae. Standard length was selected as the best size parameter for fish since the caudal fin is so frequently damaged in specimens trawled from mesopelagic depths. However, pre-anal fin length (PAFL) was used instead of SL for grenadiers (*Albatrossia pectoralis*) and (*Coryphaenoides cinereus*) following the recommended standard for the Macrouridae [42,43]. Differences between left and right otoliths are rare and when reported are small [44,28] or suspect due to small sample sizes [19]. In our study, we investigated differences between left and right otoliths only if the *R* ^2^ regression value was less than 0.90, and in the 10 species for which this was the case, we calculated separate regressions for both left and right otoliths. Potential differences between the regressions were checked by t-test and in all 10 cases no significant differences (*P*≤0.05) were indicated, so a single regression was employed.

Squid regressions were calculated using lower beak rostral length (LRL) or upper beak rostral length (URL) relative to dorsal mantle length (DML) or pen length (PL) and weight (WT). Both LRL and URL are defined as the length of the beak cutting edge between the rostral tip and the notch at the base of the wing insertion [30]. We used dorsal mantle length as the best measure of overall body size for squid following the prevailing standard [30]. The length of the pen, or gladius, is a very close approximation to dorsal mantle length and in samples with damaged mantle margins, we substituted PL for DML [45].

Depending on size, fish otolith and squid beak measurements were made with either optical micrometer or vernier calipers to the nearest 0.1 mm. Fish and squid were weighed to the nearest 0.1 g. With the exception of 5 cephalopod species, the relationship between hard part measurements to body length was best determined by least-squares linear regression function *y* = *ax*+*b*. For cephalopods *Eogonatus tinro*, *Gonatus berryi*, *Gonatus* sp. Z, *Chiroteuthis calyx* and *Taonius borealis* the LRL to DML or PL relationships were nonlinear and in these cases, we adopted the equation *y* = *ax* ^b^. The length-weight relationships for both squid and fish were determined using a least-squares regression of the log of the length and weight with subsequent transformation back to arithmetic units and presented as the function *y* = *ax* ^b^ Transformation back to arithmetic units may result in underestimating weight, however these errors are typically small [46].

### Proximate composition and energetic analysis

Proximate composition analyses were conducted on 23 species of fish and 9 species of squid. The energetic potential of prey can vary with region and season of collection as well as size (age) of specimen. Consequently, samples analyzed for proximate composition and energetic value were collected within a very narrow seasonal and temporal band in a localized area of the southeastern Bering Sea between 53°–56°N and 166°–170°W during May 15–22, 1999 and 2000 [41]. Body size and in some cases, reproductive condition served as our proxy for assigning individual age categories of juvenile (JUV), sub-adult (SA) or adult (A) in the laboratory. Samples were frozen (-40°F) in water immediately following collection at sea and then transferred to a -20°F freezer in the laboratory. Body lengths and weights were measured on pristine near frozen samples then organized by species into biologically significant size-stratified groups (JUV, SA, A) prior to refreezing and storage for up to two years preceding eventual full thawing for energetic analyses.

Whole frozen samples were thawed at the analytical laboratory (Food Products Laboratory Inc., 12003 Ainsworth Circle, Suite 105, Portland, OR 97220) prior to homogenization in a blender either singly or by species within similar body size groups. Excess water retained in squid body cavities was drained after thawing to avoid variation in moisture content values. Three gram portions of homogenate were sampled for proximate composition analysis according to the Association of Analytical Chemists (AOAC) recommended methods [47]. Duplicate samples and standard reference samples were run as quality control measures for each analysis. Samples were reanalyzed if the deviation between duplicates was greater than 15% of the mean or if the standard reference sample was not within 2.5% (or 1.2% for ash) of the derived value.

A test of distillation efficiency during protein analysis was run with ammonium sulfate. If ammonium sulfate recovery was less than 95% the samples were retested. Protein was analyzed using the Kjeldhal method [47] and the nitrogen produced was converted to percent protein with a conversion factor of 5.65. Lipid values were obtained through acid hydrolysis [47]. Moisture content (or percent moisture loss) was determined by heating samples in an oven at 130°C for two hours and then subtracting the resulting dry weight from the original wet weight [47]. Ash content, a measure of vitamins and minerals in animal tissue, was determined by combusting samples at 550°C for up to 12 hours then measuring resulting weight loss [47]. Carbohydrates are calculated as the residual number after the measured values (which are expected to add to 100%) of lipid, protein, moisture and ash are subtracted from 100. As such, carbohydrates represent the additive error inherent in each separate proximate value which is generally less than 2% or, as a measure of quality control, the samples are re-run. Carbohydrate values are not reported here since in addition to negligible error rates, fish and squid have little or no carbohydrates [48]. Energy density was calculated in calories (cal/100g) from proximate composition by multiplying the wet weight values of lipid and protein by their energy equivalents, 9.5 and 5.65, respectively. Neither ash nor moisture has caloric value and carbohydrates have a minimal effect on caloric measurements [47].

### Ethics

Fish and squid were collected for research purposes only from standard annual bottom trawl surveys and a pilot midwater trawl survey conducted by the National Oceanic and Atmospheric Administration (NOAA) Alaska Fisheries Science Center (AFSC; Seattle, Washington) groundfish assessment program. Collection of biological data in the US Exclusive Economic Zone by federal scientists to support fishery research is granted by the Magnuson—Stevens Fishery Conservation and Management Act. No protected species were sampled during the course of this study.

## Results and Discussion

Regression formulae, proximate composition values and energy (caloric) calculations are tabulated for direct application to predator diet studies and ecosystem modeling (Tables 1–4).

**Table 1:** Fish length and weight regression equations. Otolith height (OH), otolith length (OL) or dentary anterior tooth length (DATL) were measured (mm) and regressed on standard length (SL) or pre-anal fin length. Standard length or PAFL were regressed on weight (WT) (g).

Species	Regression	N	R^2^	SE	SE	Min (mm)	Max (mm)	Avg (mm)
				SE (slope)	SE (intercept)	(mm/g)	(mm/g)	(mm/g)
**Bathylagidae**								
*Bathylagus pacificus*	SL = 43.06 OL—37.793	212	0.90	0.975	3.816	1.9	6.0	3.82
	SL = 113.10 OH—61.372	212	0.83	3.55	6.01	0.9	2.3	1.66
	WT = 0.00000116 SL ^3.377^	230	0.98	0.031	0.153	49	220	136.6
*Leuroglossus schmidti*	SL = 52.57 OL—28.522	259	0.86	1.32	3.261	1.3	3.2	2.5
	SL = 88.235 OH ^1.242^	259	0.77	0.042	0.007	0.5	1.5	1.1
	WT = 0.000000531 SL ^3.523^	703	0.97	0.021	0.101	40	152	110.3
*Lipolagus ochotensis*	SL = 35.34 OL—18.243	106	0.76	1.97	6.663	1.7	2.6	3.41
	WT = 0.000002973 SL ^3.216^	235	0.94	0.054	0.248	70	146	102.9
*Pseudobathylagus milleri*	SL = 79.78 OL—89.140	170	0.72	3.841	10.389	2.1	3.7	2.68
	SL = 141.79 OH—86.14	170	0.65	7.941	11.911	1.1	1.9	1.49
	WT = 0.00000175 SL ^3.377^	75	0.97	0.068	0.333	72	190	133.7
**Opisthoproctidae**								
*Macropinna microstoma*	SL = 40.70 OH—74.099	79	0.90	1.53	6.658	2.9	5.8	4.30
	WT = 0.00002779 SL ^2.997^	63	0.96	0.080	0.365	51	153	99.8
**Gonostomatidae**								
*Sigmops gracilis*	WT = 0.00000007476 SL ^3.764^	40	0.84	0.268	1.305	113	160	130.4
**Stomiidae**								
*Chauliodus macouni*	SL = 11.33 DATL + 19.446	547	0.91	0.157	1.856	3.9	18.5	11.35
	WT = 0.00000004966 SL ^3.843^	547	0.97	0.074	0.390	115	310	196.2
**Scopelarchidae**								
*Benthalbella dentata*	SL = 59.17 OL + 14.598	41	0.86	3.838	10.736	1.7	4.3	2.8
	WT = 0.0000002387 SL ^3.618^	35	0.97	0.104	0.540	125	250	183.6
**Myctophidae**								
*Diaphus theta*	SL = 40.28 OH—25.54	241	0.94	0.641	1.524	1.4	3.2	2.3
	WT = 0.00001005 SL ^3.146^	332	0.99	0.019	0.084	33	105	78.5
*Lampanyctus jordani*	SL = 46.58 OH—6.36	154	0.81	1.843	4.747	1.6	3.2	2.6
	WT = 0.000000418 SL ^3.752^	398	0.91	0.243	0.280	85	143	118.5
*Nannobrachium regale*	SL = 79.61OH—22.42	124	0.82	3.369	6.920	1.3	3.0	2.0
	WT = 0.00000104 SL ^3.454^	180	0.96	0.053	0.262	85.0	200	143.9
*Protomyctophum thompsoni*	SL = 22.87 OH—4.545	36	0.81	1.922	4.684	2.0	2.7	2.4
	WT = 0.0000389 SL ^2.805^	63	0.90	0.119	0.469	36	69	51.5
*Stenobrachius leucopsarus*	SL = 43.63 OH—0.829	380	0.94	0.578	1.069	1.8	2.7	1.8
	WT = 0.00000656 SL ^3.121^	1221	0.98	0.011	0.047	31	120	68.8
*Stenobrachius nannochir*	SL = 44.65 OH + 2.17	342	0.91	0.748	1.505	1.0	2.7	2.0
	WT = 0.00000693 SL ^3.082^	305	0.98	0.022	0.097	35	130	85.7
**Macrouridae**								
*Albatrossia pectoralis* ^*a*^	PAFL = 15.64 OL—21.71	122	0.96	0.298	0.232	3.4	28.8	10.17
	WT = 0.0000237 PAFL ^3.310^	120	0.99	0.038	0.185	39	486	137.2
*Coryphaenoides cinereus* ^*a*^	PAFL = 21.44 OL—13.75	281	0.91	0.529*	2.898*	1.7*	8.4*	5.22*
	WT = 0.00000107 PAFL ^3.210^	281	0.99	0.021*	0.094*	23	164	98*
**Melamphaidae**								
*Melamphaes lugubris*	SL = 14.72 OL—12.858	206	0.87	0.395	2.437	4.1	7.5	6.14
	SL = 29.68 OH -14.860	206	0.86	0.855	2.677	2.1	3.8	3.11
	WT = 0.00005935 SL ^2.829^	255	0.93	0.049	0.218	50	109	81.8
*Poromitra crassiceps*	SL = 27.77 OL—11.571	140	0.63	1.837	7.449	3.0	5.1	4.03
	WT = 0.00002099 SL ^2.984^	613	0.88	0.044	0.205	61	140	105.0
**Zoarcidae**								
*Lycodapus fierasfer*	WT = 0.000001102 SL ^3.245^	209	0.96	0.047	0.221	62	156	110.6

^a^ Regression data adapted from Walker et al. 2002 [27].

**Table 2:** Cephalopod length and weight regression equations. Lower beak rostral length (LRL) and upper beak rostral length (URL) were measured (mm) and regressed on dorsal mantle length (DML) or pen length (PL). Dorsal mantle length or PL was regressed on weight (WT) (g).

Species	Regression	N	R^2^	SE (slope)	SE (intercept)	Min (mm/g)	Max (mm/g)	Avg (mm/g)
**Gonatidae**								
*Berryteuthis anonychus*	DML = 38.67 LRL + 21.18	73	0.88	1.714	2.65	0.8	2.4	1.52
	WT = 0.00124 DML2.182	33	0.96	0.08	0.343	43	108	73.7
*Berryteuthis magister*	DML = 40.43 LRL—2.502	275	0.99	0.298	0.896	0.45	10	2.35
	DML = 45.47 URL—0.72	121	0.97	0.697	1.996	0.6	6.3	2.45
	WT = 0.00008101 DML 2.816	817	0.99	0.01	0.035	17	386	84.9
*Eogonatus tinro*	PL = 17.814 LRL 1.303	693	0.91	0.016	0.017	0.95	6.3	2.99
	WT = 0.000222 PL 2.632	1039	0.95	0.018	0.075	24	230	69.9
*Gonatopsis borealis*	DML = 38.14 LRL + 2.11	482	0.99	0.196	0.538	0.5	4.7	2.37
*(northern form)*	DML = 42.01 URL + 0.26	88	0.97	0.76	1.53	0.7	4.1	1.85
	WT = 0.00007142 DML 2.872	1069	0.99	0.007	0.03	25	183	95.6
*Gonatopsis / Berryteuthis* ^a^	DML = 39.37 LRL—0.50	757	0.98	0.179	0.507	0.45	10	2.37
	WT = 0.01561 DML 2.872	1676	0.99	0.006	0.011	17	386	89.1
*Gonatus berryi*	PL = 11.023 LRL 1.571	74	0.94	0.048	0.061	1.8	5.6	3.55
	WT = 0.000254 PL2.592	58	0.97	0.064	0.288	26	203	98.9
*Gonatus middendorffi*	DML = 47.51 LRL + 1.72	79	0.98	0.7	1.756	1.1	8	2.1
	WT = 0.000139 DML 2.552	58	0.98	0.044	0.195	46	125	83.5
*Gonatus onyx*	PL = 24.65 LRL + 4.30	210	0.92	0.493	0.983	1.05	4.2	1.94
	WT = 0.000111 PL2.732	209	0.93	0.05	0.195	27	108	50
*Gonatus pyros*	PL = 15.81 LRL + 9.03	196	0.94	0.283	0.675	1	4.8	2.25
	WT = 0.000269 PL2.595	136	0.92	0.065	0.242	20	90	41.9
*Gonatus sp*. *Z*	PL = 17.637 LRL 1.129	90	0.87	0.047	0.065	1.5	6.2	3.96
	WT = 0.000116 PL2.777	78	0.97	0.054	0.249	31	194	101
**Chiroteuthidae**								
*Chiroteuthis calyx*	DML = 11.473 LRL 1.508	42	0.86	0.096	0.151	2.1	6.1	4.86
	WT = 0.00147 DML2.325	31	0.96	0.091	0.445	54	205	140.2
**Cranchiidae**								
*Galiteuthis phyllura*	DML = 94.35 LRL—2.52	105	0.94	2.24	5.18	0.7	6	2.13
	WT = 0.000125 DML2.145	99	0.94	0.053	0.273	47	372	193
*Taonius borealis*	DML = 75.944 LRL 0.735	203	0.93	0.015	0.018	1.2	8.6	3.4
	WT = 0.000000135 DML3.595	145	0.94	0.075	0.393	82	445	195.1

^a^ adapted from Gudmundson et al. [49].

**Table 3:** Fish proximate analyses. Maturity status was approximated by body size and classified as adult (A), sub-adult (SA) or juvenile (JUV). All lengths are standard length except where noted.

Species	Individuals	Composites	Total weight (g)	Length range (mm)	Mean length (mm)	Maturity status	%Fat range	%Fat mean	%Protein range	%Protein mean	%Moisture range	%Moisture mean	%Ash range	%Ash mean	Energy Content range (cal/100g)	Energy Content mean (cal/100g)
**Microstomatidae**																
*Nansenia Candida*	2	1	152	210–220	215	A	19.0	-	12.1	-	70	-	1.1	-	248.9	
**Bathylagidae**	4	1	182	139–178	157	SA	6.2	-	8.7	-	85.9	-	0.7	-	108.1	
*Bathylagus milleri*	70	3	735	85–135	107	SA	10.2–11.2	10.7	8.2–8.9	8.5	77.5–79.2	78	1.0–1.2	1	144.9–156.7	149.5
*Bathylagus ochotensis*	79	5	1333	100–148	129	SA	2.7–3.9	3.3	4.9–8.2	6.2	87.8–88.8	88	0.9–1.5	1	57.1–73.6	66.4
	53	8	2104	151–200	171	SA	2.6–7.2	3.9	4.1–10.3	7.5	80.6–88.9	86	0.9–1.7	1	57.4–122.6	79.1
*Leuroglossus schmidti*	163	7	1877	103–146	120	SA	12.4–16.4	14.1	7.9–10.5	9.5	71.2–79.2	76	0.5–1.3	1	164.3–214.2	187.4
**Opisthoproctidae**																
*Macropinna microstoma*	10	1	272	73–136	104	SA	4.8	-	9.6	-	84.7	-	0.9	-	99.8	-
**Gonostomatidae**																
*Sigmops gracilis*	26	1	173	117–144	129	SA	18.0	-	11.3	-	69.2	-	0.8	-	234.9	-
**Stomiidae**																
*Chauliodus macouni*	9	2	506	167–279	248	SA	7.2–7.8	7.5	10.0–10.8	10.4	77.7–80.9	79	0.9–1.4	1	129.4–130.6	130.0
*Tactostoma macropus*	3	1	345	285–370	333	SA	10.3	-	10.3	-	74.7	-	0.7	-	156.1	-
**Scopelarchidae**																
*Benthalbella dentata*	6	1	163	125–198	165	SA	18.8	-	13.1	-	67	-	1.1	-	252.6	-
**Notosudidae**																
*Scopelosaurus harryi*	2	1	146	237–255	246	A	8.2	-	10.1	-	80.5	-	1.2	-	135.0	-
**Myctophidae**																
*Diaphus theta*	48	3	717	85–100	91	A	23.4–25.2	24.2	10.6–10.8	10.7	63.3–64.1	64	1.2–1.5	1	288.3–299.3	289.8
*Lampanyctus jordani*	30	4	964	115–135	127	A	19.3–26.9	22.0	11.4–12.5	12.2	65.3–68.3	67	1.2–1.6	1	254.0–325.6	278.0
*Nannobrachium regale*	15	2	507	133–180	148	SA	11.5–12.7	12.1	11.5–12.1	11.8	74.2–75.6	75	1.0–1.1	1	174.2–189.0	181.6
*Protomyctophum thompsoni*	42	1	79	47–58	52	A	7.9	-	11.9	-	78.2	-	2.0	-	142.3	-
*Stenobrachius leucopsarus*	114	1	304	38–78	61	JUV	13.7	-	10.7	-	71.5	-	1.4	-	190.6	-
	153	5	1414	80–120	93	A	18.1–20.1	19.1	11.3–13.9	12.6	64.7–68.1	66	1.2–1.6	1	244.1–263.8	253
*Stenobrachius nannochir*	208	5	1414	70–115	90	A	16.0–18.2	17.0	10.0–12.6	11.1	67.5–72.4	70	1.4–1.8	2	212.5–244.1	223.6
**Macrouridae**																
*Albatrossia pectoralis*	2	1	176	91–95^a^	93^a^	JUV	3.7	-	8.9	-	86.5	-	0.9	-	85.4	-
	1	1	413	-	148^a^	JUV	7.3	-	12.2	-	79.6	-	0.9	-	138.3	-
**Oneirodidae**																
*Oneirodes thompsoni*	3	1	328	90–116	103	SA	2.7	-	8.3	-	87.5	-	1.4	-	75.6	-
**Melamphaidae**																
*Melamphaes lugubris*	47	3	764	73–95	86	A	31.6–33.2	32.3	9.7–10.7	10.2	56.0–60.5	58	2.1–2.3	2	358.4–375.9	365.0
*Poromitra crassiceps*	45	4	1066	101–128	109	A	15.3–18.0	16.5	8.8–10.6	9.8	71.3–76.7	75	1.2–1.6	2	200.8–228.6	211.7
**Zoarcidae**																
*Bothrocara brunneum*	9	2	553	186–361	294	JUV	0.8–1.0	0.9	6.8–10.6	8.7	84.2–86.7	86	1.7–2.2	2.0	46.2–69.4	57.8
*Lycodapus fierasfer*	43	1	256	92–156	119	SA	6.1	-	9.1	-	83.7	-	1.3	-	109.4	-

^a^ length records based on pre-anal fin length.

**Table 4:** Squid proximate analyses. Maturity status was based on reproductive condition and body size and classified as juvenile (Juv) or sub-adult (SA). All lengths are dorsal mantle length except where noted.

Species	Individuals	Composite	Total weight (g)	Length range (mm)	Mean length (mm)	Maturity status	%Fat range	%Fat mean	%Protein range	%Protein mean	%Moisture range	%Moisture mean	%Ash range	%Ash mean	Energy Content range (cal/100g)	Energy Content mean (cal/100g)
**Gonatidae**																
*Berryteuthis anonychus*	11	1	143	65–75	71	JUV	4.4	-	10.6	-	84.7	-	0.3	-	101.7	-
*Berryteuthis magister*	44	1	260	45–64	53	JUV	3.3	-	10.3	-	86.0	-	0.5	-	89.6	-
	6	5	1193	144–212	180	JUV	2.4–3.9	3.8	5.8–12.3	8.6	83.4–86.0	84.9	0.6–0.7	0.7	60.0–106.6	84.4
	6	6	2253	213–238	229	SA	7.0–9.7	8.6	11.5–12.4	12.0	76.5–80.6	78.6	0.6–0.7	0.7	131.4–160.3	148.5
*Eogonatus tinro*	110	6	1519	49–88^a^	65^a^	JUV	10.2–13.1	12	7.6–9.1	8.4	71.7–81.7	80.0	0.3–0.5	0.3	142.7–188.5	160.0
	5	3	877	147–198^a^	170^a^	SA	10.5–13.4	12.0	8.0–8.2	8.1	76.2–78.9	78.0	0.3–1.3	0.8	145.0–173.6	160.0
*Gonatopsis borealis*	53	1	261	32–76	45	JUV	1.9	-	9.1	-	88.6	-	0.3	-	69.5	-
	29	6	1342	83–134	104	JUV	1.8–3.3	2.7	9.7–14.0	11.7	82.8–88.0	85.6	0.3–0.5	0.4	73.0–110.5	91.0
	20	9	2421	134–179	150	SA	2.1–6.4	3.9	9.7–13.8	10.8	85.7–86.8	84.7	0.3–0.8	0.4	78.2–138.8	98.4
*Gonatus berryi*	5	1	148	75–125^a^	102^a^	JUV	8.9	-	6.2	-	82.3	-	0.2	-	119.6	-
**Chirote uthidae**																
*Chiroteuthis calyx*	3	3	382	190–202	197	-	4.1–6.8	5.0	4.9–8.8	6.6	84.9–88.2	86.6	0.4–1.2	0.8	66.7–99.1	85.1
**Cranchiidae**																
*Galiteuthis phyllura*	13	1	336	215–372	283	JUV	3.3	-	9.3	-	87.8	-	0.4	-	84.0	-
*Taonius borealis*	6	3	1329	215–482	337	JUV	1.0–5.7	4.0	6.9–11.0	8.9	84.5–89.0	86.4	0.4–1.2	0.8	48.5–112.5	88.3

^a^ length records based on pen length.

A regression formula for one species of fish (*Diaphus theta*) presented here has been evaluated in the past [22] as have formulae for the gonatid squid: *Berryteuthis anonychus*, *Berryteuthis magister*, *Gonatopsis borealis*, *Gonatus middendorffi* and *Gonatus onyx* [30,31,49,50]. We present new regression formulae for these based on enhanced sample sizes and body size ranges with consequently tighter *R* ^2^ values than those previously published. All energetic data presented here are new to the published literature in the region of collection.

**Fig 1:**
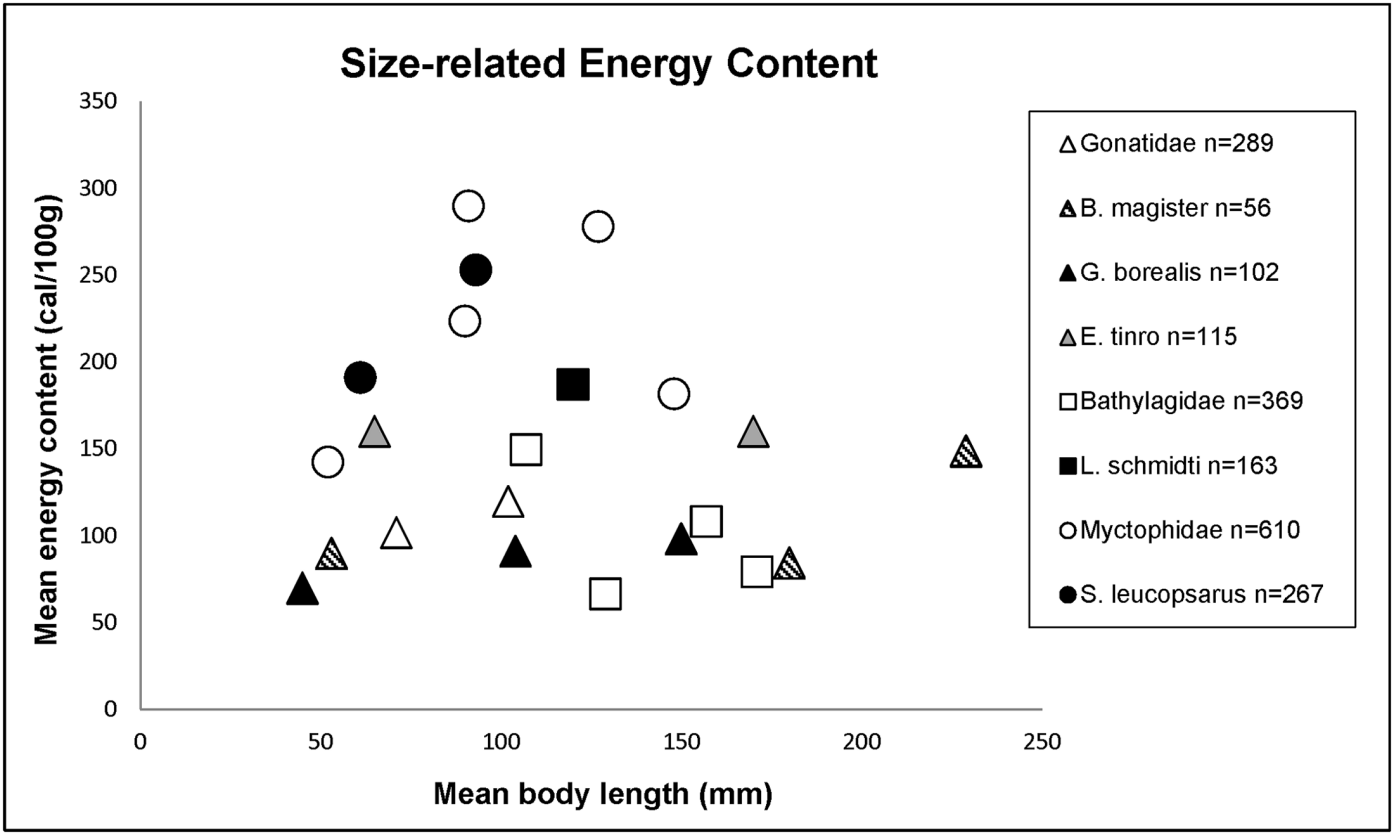
Size-related energetic content. Relative size related energy content of dominant fish and squid families and species caught in Bering Sea research trawls during 1999 and 2000.

It is notable that the families of fish (Myctophidae, Bathylagidae) and squid (Gonatidae) that dominated our trawl catch [41] also dominate the mesopelagic portion of marine bird and mammal diets in the Bering Sea and North Pacific Ocean [1,51,10]. The numerically dominant species of fish (*Stenobrachius leucopsarus*, *Leuroglossus schmidti*) and squid (*G*. *borealis*, *B*. *magister*) that were caught also rank numerically highest in predator diets compared to other family members and were either comparable to, or ranked energetically highest among family mean values in this study (Fig 1; Tables 3 and 4).

**Fig 2:**
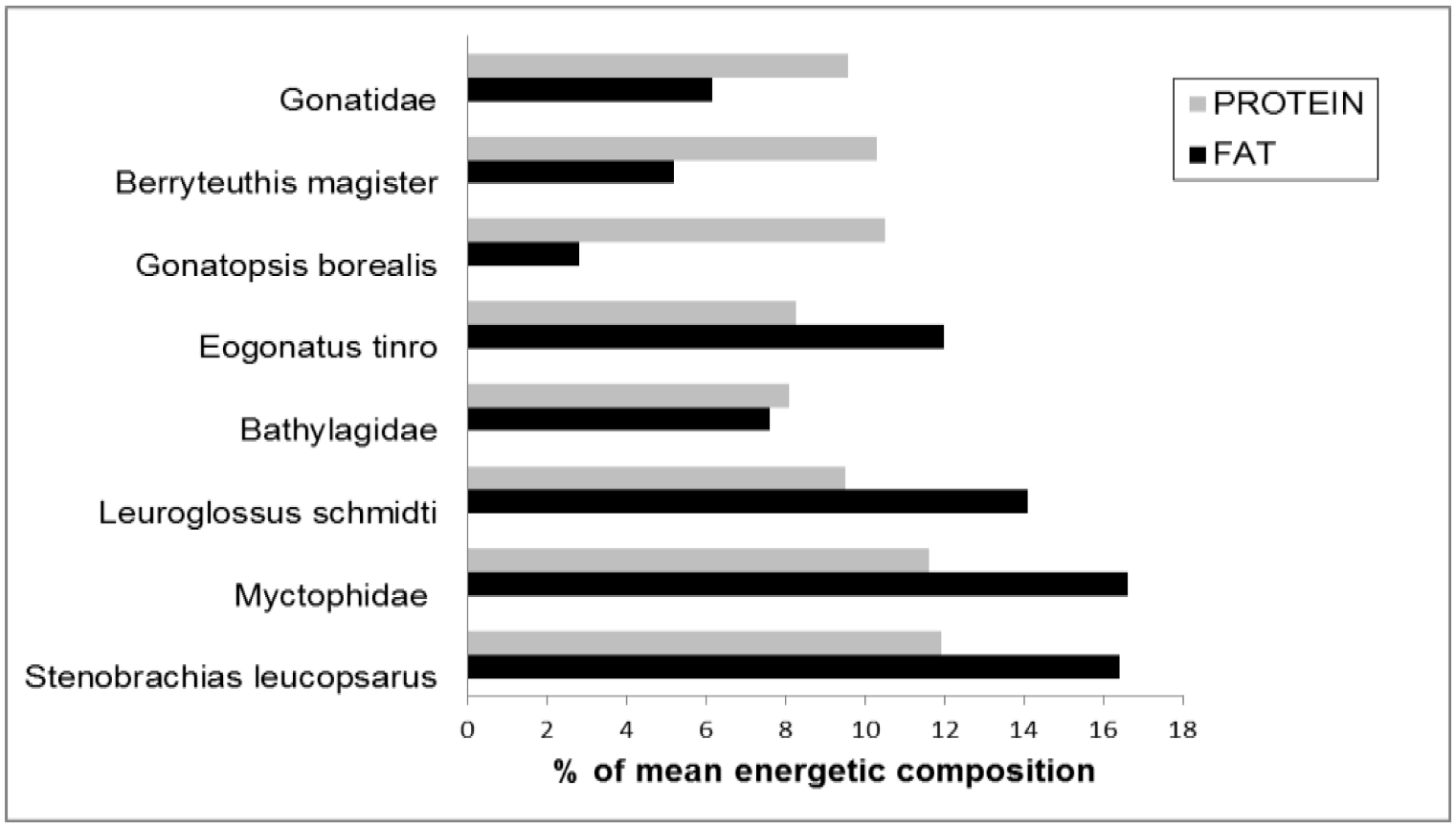
Percent contribution of fat and protein to energetic composition. Relative contribution of fat and protein to energy content of dominant fish and squid families and species caught in Bering Sea research trawls during 1999 and 2000.

The Myctophidae were significantly (*P*≤ 0.05) higher in mean energy, fat and protein values than the Bathylagidae or the Gonatidae (Figs 1 and 2; Tables 3 and 4). Two exceptions to family patterns among fishes were the myctophid *Protomyctophum thompsoni* (Table 3) and the bathylagid *L*. *schmidti* with respectively lower and higher caloric value compared to the rest of their families (Fig 1; Table 3). Sub-adult *L*. *schmidti* were comparable in proximate composition and energy value to juvenile *S*. *leucopsarus*, a species with high measures of protein, fat and subsequent energy values that are typical of the myctophid family (Figs 1 and 2; Table 3). Gonatid squid were significantly (*P*≤ 0.05) higher in protein than Bathylagidae but, generally lower in fat and as a result, comparable in overall energy values. Eogonatus tinro was an exception among the Gonatidae with significantly higher fat and lower protein values making it comparable to *L*. *schmidti*, and contributing towards overall energy values that are the highest among the Gonatidae.(Figs 1 and 2; Tables 3 and 4).

Myctophid fishes provide more energy in terms of both fat and protein than either bathylagid fishes or gonatid squids, but these results may be variably influenced by specimen age (as estimated by body size) and reproductive condition which was determined for only a subset of all taxa sampled in this study (Fig 1; Tables 3 and 4). In cases where sample sizes were large enough for analysis of proximate composition according to body size and reproductive condition, we found that energy values increased with age, as determined by body length, for all but one gonatid squid species (*E*. *tinro*) (Fig 1; Table 4). *Eogonatus tinro* is significantly (*P*≤ 0.05) higher in measures of fat and energy (but, not protein) than any other member of the gonatid family in both juvenile and sub-adult stages (Table 4). This could be a factor of sampling or sample size and it should be noted that *B*. *magister* does not increase in energy value until reaching a DML of over 20 cm (Fig 1; Table 4).

This paper is meant to serve as a resource guide for those wishing to incorporate mesopelagic fish and squid body size regression formulae and size-related energetic value in their own work.

We have accounted for several of the variables that influence intraspecific energy composition. Large samples were collected in the same place at the same time of year and were evaluated by body size as a proxy for age wherever possible. If not for limited life history information on most mesopelagic species, our analysis would have been further improved by directly aging each individual sample since interspecific energetic value is known to increase by size within age categories, particularly for batch spawners [36]. We emphasize the importance of evaluating fat and protein separately by size/age category wherever possible for several reasons: 1) both protein and fat drive energetic value; 2) intraspecific protein and fat values vary with relative life history stages and collection location [34,36]; and 3) protein and fat are variably important to predators at different life stages [52]. Ultimately, age-related proximate composition values are important variables in describing the energetic map and energy flux in the world’s oceans.
