# Correction: Body Size Regression Formulae, Proximate Composition and Energy Density of Eastern Bering Sea Mesopelagic Fish and Squid

**DOI:** 10.1371/journal.pone.0159353

**Published:** 2016-07-12

**Authors:** Elizabeth H. Sinclair, William A. Walker, James R. Thomason

There are formatting errors in Table 2. The exponential equations should appear with superscript formatting. Please see the correct Table 2 and its caption here.

**Table 2:** Cephalopod length and weight regression equations. Lower beak rostral length (LRL) and upper beak rostral length (URL) were measured (mm) and regressed on dorsal mantle length (DML) or pen length (PL). Dorsal mantle length or PL was regressed on weight (WT) (g).

Species	Regression	N	R^2^	SE (slope)	SE (intercept)	Min (mm/g)	Max (mm/g)	Avg (mm/g)
**Gonatidae**								
*Berryteuthis anonychus*	DML = 38.67 LRL + 21.18	73	0.88	1.714	2.65	0.8	2.4	1.52
	WT = 0.00124 DML^2.182^	33	0.96	0.08	0.343	43	108	73.7
*Berryteuthis magister*	DML = 40.43 LRL-2.502	275	0.99	0.298	0.896	0.45	10	2.35
	DML = 45.47 URL-0.72	121	0.97	0.697	1.996	0.6	6.3	2.45
	WT = 0.00008101 DML ^2.816^	817	0.99	0.01	0.035	17	386	84.9
*Eogonatus tinro*	PL = 17.814 LRL ^1.303^	693	0.91	0.016	0.017	0.95	6.3	2.99
	WT = 0.000222 PL ^2.632^	1039	0.95	0.018	0.075	24	230	69.9
*Gonatopsis borealis*	DML = 38.14 LRL + 2.11	482	0.99	0.196	0.538	0.5	4.7	2.37
(northern form)	DML = 42.01 URL + 0.26	88	0.97	0.76	1.53	0.7	4.1	1.85
	WT = 0.00007142 DML ^2.872^	1069	0.99	0.007	0.03	25	183	95.6
*Gonatopsis / Berryteuthis*^a^	DML = 39.37 LRL-0.50	757	0.98	0.179	0.507	0.45	10	2.37
	WT = 0.01561 DML ^2.872^	1676	0.99	0.006	0.011	17	386	89.1
*Gonatus berryi*	PL = 11.023 LRL ^1.571^	74	0.94	0.048	0.061	1.8	5.6	3.55
	WT = 0.000254 PL^2.592^	58	0.97	0.064	0.288	26	203	98.9
*Gonatus middendorffi*	DML = 47.51 LRL + 1.72	79	0.98	0.7	1.756	1.1	8	2.1
	WT = 0.000139 DML ^2.552^	58	0.98	0.044	0.195	46	125	83.5
*Gonatus onyx*	PL = 24.65 LRL + 4.30	210	0.92	0.493	0.983	1.05	4.2	1.94
	WT = 0.000111 PL^2.732^	209	0.93	0.05	0.195	27	108	50
*Gonatus pyros*	PL = 15.81 LRL + 9.03	196	0.94	0.283	0.675	1	4.8	2.25
	WT = 0.000269 PL^2.595^	136	0.92	0.065	0.242	20	90	41.9
*Gonatus* sp. *Z*	PL = 17.637 LRL ^1.129^	90	0.87	0.047	0.065	1.5	6.2	3.96
	WT = 0.000116 PL^2.777^	78	0.97	0.054	0.249	31	194	101
**Chiroteuthidae**								
*Chiroteuthis calyx*	DML = 11.473 LRL ^1.508^	42	0.86	0.096	0.151	2.1	6.1	4.86
	WT = 0.00147 DML^2.325^	31	0.96	0.091	0.445	54	205	140.2
**Cranchiidae**								
*Galiteuthis phyllura*	DML = 94.35 LRL-2.52	105	0.94	2.24	5.18	0.7	6	2.13
	WT = 0.000125 DML^2.145^	99	0.94	0.053	0.273	47	372	193
*Taonius borealis*	DML = 75.944 LRL ^0.735^	203	0.93	0.015	0.018	1.2	8.6	3.4
	WT = 0.000000135 DML^3.595^	145	0.94	0.075	0.393	82	445	195.1

^a^ adapted from Gudmundson et al. [49].
